# Evaluating the Reach, Usage, Human Support Needs, and Clinical Outcomes of Digital Parent Training for Child Oppositional Defiant Disorder Before and During Wartime: Longitudinal Study

**DOI:** 10.2196/80420

**Published:** 2025-12-22

**Authors:** Amit Baumel, Or Brandes, Chen R Saar

**Affiliations:** 1 Department of Community Mental Health University of Haifa Haifa Israel

**Keywords:** parent training, crisis, behavior problems, usage, oppositional defiant disorder, wartime, digital health

## Abstract

**Background:**

Digital parent training programs (DPTs) have emerged as a scalable solution for treating childhood oppositional defiant disorder (ODD), offering remote access and reduced barriers to care. However, there is limited data on their potential to reach untreated populations and their effectiveness during times of crisis, such as war.

**Objective:**

This study aimed to evaluate the reach, usage patterns, human support needs, and clinical outcomes of a fully remote guided DPT for child ODD, comparing 2 cohorts treated before and during wartime in Israel.

**Methods:**

Parents of children with ODD were enrolled in a human-supported DPT, with 25 families recruited before and 30 during wartime. Data included self-reported questionnaires (measured before-, postintervention, and 3 months after the end of the intervention), platform usage metrics, and clinician assessments.

**Results:**

Most families (62%, 34/55) had not previously received any intervention for their child’s behavior problems. Significant self-reported improvements in child behavior (Cohen *d*≥0.79) and parenting practices (0.39≤Cohen *d*≤0.87) were found post intervention. On average, families engaged with the program for 138.6 minutes across 31.4 unique logins, supported by 38.8 minutes of human interaction, primarily via messaging. During wartime, parents completed onboarding significantly faster (15.70 days vs 31.36 days) and were more likely to complete the critical “overcoming disobedience” phase (27/30, 90% vs 17/25, 68%). However, while self-reported changes were similar, clinician-rated recovery from ODD was marginally lower during wartime (13/30, 43% vs 17/25, 68%).

**Conclusions:**

DPTs present an acceptable avenue for care that could reach parents who have not sought treatment through traditional channels. However, this study’s results suggest that their clinical effectiveness may be lower under extreme stress conditions such as wartime, underscoring the need for future studies in this area.

## Introduction

Oppositional defiant disorder (ODD) is among the most prevalent types of mental disorders affecting young children, and it is crucial to treat it at an early onset [1]. However, the provision of child mental health services is limited [2]. In Israel, the wait times for publicly administered face-to-face mental health interventions are very long, with an ongoing gap between supply and demand that is not easily closed [3]. To address barriers related to service delivery in the treatment of behavior problems, digital parent training programs (DPTs) aimed at treating childhood disruptive behaviors have emerged over the last decade [4-6]. These interventions have demonstrated efficacy in meta-analyses [7-9], with trials reporting at least moderate effect size improvements in child behaviors for guided DPTs (Cohen *d*>0.50) [7,9,10].

Although DPTs appear to offer a feasible way to increase access to care, it is unclear the extent to which these interventions reduce the mental health treatment gap by reaching parents who have not previously sought treatment for their child’s condition. Furthermore, it is uncertain how much human support is needed as an adjunct to DPT usage, especially when the program’s design incorporates automated features that potentially reduce the need for human support. Our own study of DPTs for child behavior problems showed that, compared to a standard format, an enhanced intervention in terms of its design increased user engagement (2.5 times higher completion rate) and program efficacy in reducing child behavior problems (Cohen *d*=0.43-0.54) in a self-guided condition [11]. However, the degree of human support required when deploying such interventions remains unclear.

In addition, this study began before the war in Israel (which began on October 7, 2023) and continued during wartime. This unexpected war resulted in a deterioration in mental health and a profound need for mental health support for the whole population [12]. For this reason, we sought to use our data to answer questions relating to the impact of a conflict setting on the acceptability and effectiveness of DPTs. There is a significant gap in the literature, with very limited evidence on whether the use of digital mental health interventions for child routine care differs under conditions of war. A notable study examined enrollment and completion rates of a nationwide guided DPT for children with disruptive behavior before and during the COVID‑19 pandemic [13]. In the study, program enrollment did not change significantly under COVID-19 restrictions, while the completion rate actually increased. It remains unclear whether the usage of guided DPTs for child behavior problems increases during wartime, as observed during COVID-19, and what their effectiveness is in terms of clinical outcomes, an area that is yet to be explored.

The main aim of this study was to evaluate the potential deployment of a DPT for child behavior problems as a scalable form of care. In particular, our study (1) examined the extent to which a remote onboarding procedure, including reaching people through social media, could reach parents who had not previously sought treatment for the relevant condition; (2) evaluated the timeline of remotely administered onboarding process, which included contact with a clinician; (3) evaluated the DPT’s usage patterns, human support, and effectiveness; and (4) assessed differences in reach, usage, and efficacy in the context of war, which began halfway through the study’s deployment.

## Methods

### Participants and Recruitment Procedure

#### Eligibility

Parents were eligible to participate if they met the eligibility criteria (see Textbox 1).

**Eligibility criteria.**

Inclusion criteria:

- Parents had a child between the ages of 3 and 7 years with an elevated level of behavior problems based on the Eyberg Child Behavior Inventory (ECBI; ECBI problem≥15 and ECBI intensity≥132) [14].
- The behavior problems described by the parents in an interview conducted by a licensed clinician met the criteria for oppositional defiant disorder.
- They had access to a smartphone device and a personal computer with an internet connection.

Exclusion criteria:

- Their child was taking medication or was in regular contact with a professional for behavioral or emotional problems.
- They were currently accessing parenting support elsewhere.
- Their child had been diagnosed with an intellectual disability or developmental delay.

#### Recruitment Procedure

Parents were recruited for the study from January 1, 2023, to January 10, 2024, through Facebook advertising, a method previously shown to reach broad and diverse populations across socioeconomic backgrounds in ways comparable to traditional recruitment approaches [15]. Because the program and all study materials were provided in Hebrew, the campaign specifically targeted Hebrew-speaking parents of children aged between 3 and 7 years, residing in Israel. There was a planned pause in recruitment between May 2023 and the beginning of October 2023 due to summer and holiday breaks in the education system. Recruitment was then delayed because the war began on October 7, 2023, which meant that the recruitment of the 2 cohorts (before and during wartime) took place during 2 consecutive school years.

Parents registered their interest in participation through the study home page, where they completed a brief eligibility screener as a first step. Screening, interest, and parents’ understanding of the terms of the study were confirmed through a phone call with a research assistant, followed by a link to complete the informed consent online. Parents then participated in a remote assessment with an independent clinician (author OB). Eligible participants were then referred to complete the baseline assessment. Those who completed the assessment received login details to the intervention’s platform, with complementary guidance from their assigned human supporter. Parents who were not eligible to participate were referred to local services. No compensation was provided for participation, other than access to the intervention.

### Benevolent Parenting Intervention

Multimedia Appendix 1 presents a comprehensive review of Benevolent Parenting DPT. The program is based on predefined decision rules that are either event- or time-based [16], meant to increase user engagement through effort optimization design [17] and therapeutic persuasiveness, which integrate behavior change techniques with persuasive design features specifically for digital delivery [18,19]. This framework complements the taxonomy of behavior change techniques [20] by emphasizing effective operationalization and delivery of techniques in-app (eg, prompts, self-monitoring, feedback, and adaptation), a distinction that is particularly important in digital interventions where technique presence without proper delivery yields limited impact. A self-directed version of the program has been shown in previous research to achieve a high therapeutic persuasiveness evaluation score (4.5/5) and high completion rates among parents [11].

The program incorporates common themes of evidence-based DPTs for childhood disruptive behaviors (eg, overcoming disobedience). Each theme in the program comprises a short 10-20 minute learning phase followed by a 1-2 week focusing phase. The focusing phase is designed to help the desired therapeutic activities become salient in the parents’ minds and to help them acquire skills in a nonjudgmental manner, while avoiding the burden and potential failures that may be associated with the idea of “training.” For that reason, the program incorporates the following features: (1) call to action: timely digital triggers related to the specific goals of the modules they have completed; (2) creation of salience through self-monitoring and ongoing feedback; and (3) adaptation to user state based on parents’ reports, which are used to acknowledge their success and to suggest additional actions.

Human support followed a manual based on our previous work [21,22] and included: an initial message sent when parents first logged into the program, a message following the completion of each learning and focusing phase, contacting parents via the messaging platform if disengagement was identified, response to participant-initiated messages, and a phone conversation offered following the completion of the “overcoming disobedience” module to reinforce parental efforts during the subsequent focusing phase (see Multimedia Appendix 1 for a detailed review). The supporter used a dashboard that provided information on engagement markers and participants' progress.

### Measures

Parents were assessed at 3 time points: before the intervention (baseline, T1), immediately after (postintervention, T2), and 3 months after the end of the intervention (follow-up, T3).

#### Self-Reported Measures

Child behavior problems were assessed using the Intensity and Problem subscales of the 36-item Eyberg Child Behavior Inventory (ECBI) [14,23,24] (α coefficients: ECBI Problems, α=.89 and ECBI Intensity, α=.91). Parental disciplinary behaviors in response to their child’s misbehaviors were assessed using 2 Parenting Scale (PS) subscale scores—Overreactivity (11 items) and Laxness (10 items) [25] (α coefficient: Laxness, α=.90 and Overreactivity, α=.82). Task-specific self-efficacy was assessed using items taken from the Parenting Tasks Checklist (PTC) Setting Self-efficacy (six statements) and Behavioral Self-efficacy (six statements) subscales [26] (coefficient ⍺: PTC Setting, 0.85; PTC Behavioral, 0.96). Positive parenting practices were assessed using the Alabama Parenting Questionnaire (APQ) Positive Parenting Practices subscale (six items) [27] (coefficient ⍺=0.79).

#### Clinician Assessment of ODD

A structured interview was conducted with the parents, administered by a licensed clinician (clinical child psychologist) at T1 and T2 using a secure online video connection. The clinician was trained by the study’s principal investigator (a licensed clinical psychologist with expertise in treating children and their parents) on the interview protocol. The clinician confirmed with the parents that no exclusion criteria were met (at T1) and examined whether the child met the criteria for ODD. To do so, the clinician addressed and documented the child’s symptoms of ODD (according to the Diagnostic and Statistical Manual of Mental Disorders, Fifth Edition) reported by the parents (at T1 and T2), following Pelham and colleagues’ [28,29] paradigm that demonstrated sufficient internal reliability and validity. Any questions regarding the symptoms of ODD that arose in specific cases were discussed with the study’s principal investigator before making a final decision.

#### Program Usage and Completion Rate

Measures of program usage included the number of login days, unique logins, and total time of use. We also recorded the percentage of people completing the “overcoming disobedience” module, which was deemed the main obligatory component in the intervention, and the percentage of program completion. The metrics of human support documented during the intervention delivery were message content, the occurrence of phone calls, and the time spent during each supportive contact (including the time to take notes).

### Statistical Analyses

Demographic, usage, and clinical characteristics are reported as frequencies and percentages for categorical variables, and as mean (SD) for continuous variables. Differences between the 2 study cohorts were calculated using Mann-Whitney *U* tests for continuous variables and chi-square tests for categorical variables (due to sample size limitations). Pre-post differences in outcomes over time were calculated using dependent-sample 1-tailed *t* tests for the total sample and Wilcoxon signed-rank tests within each cohort (pre- and during-wartime) to reduce reliance on normality assumptions given the modest and unequal subgroup sizes. Effect size estimates included Cohen *d* for *t* tests, the Crammer *V* for chi-square tests, and *r* for Mann–Whitney *U* and Wilcoxon tests (calculated based on the *z* value from the test divided by the total number of observations).

We determined the study sample size based on the total participant population, as we had not planned to divide the sample into 2 groups according to wartime conditions. Data on guided DPTs aimed at treating children at the *clinical* range of behavior problem symptoms point to at least moderate effect size improvement in child behavior problems (eg, child behavior problems: Cohen *d*>0.50, intent-to-treat analyses) [7,9,10]. Even when taking the lower end of this spectrum (an estimated effect size of 0.50), a sample size of 50 participants provides 0.96 power (1-tailed α=.05) to detect significant differences in the continuous variables.

We conducted intent-to-treat analyses for all reported clinical outcomes at postintervention time points. Multiple imputations (5 sets) were generated using predictive mean matching as the imputation. Sensitivity analysis was performed, showing that the statistical test results from complete case analyses were similar to the imputed analyses (completer analyses are presented in Multimedia Appendices 2 and 3). All analyses were performed using SPSS (version 27; IBM Corp).

### Ethical Considerations

The study was approved by the institutional review board (ethics committee) of the University of Haifa (342/22). Screening, interest, and parents’ understanding of the terms of the study were confirmed through a phone call with a research assistant, followed by a link to complete the informed consent online. Parents who were not eligible to participate were referred to local services. No compensation was provided for participation, other than access to the intervention.

## Results

**Figure 1:**
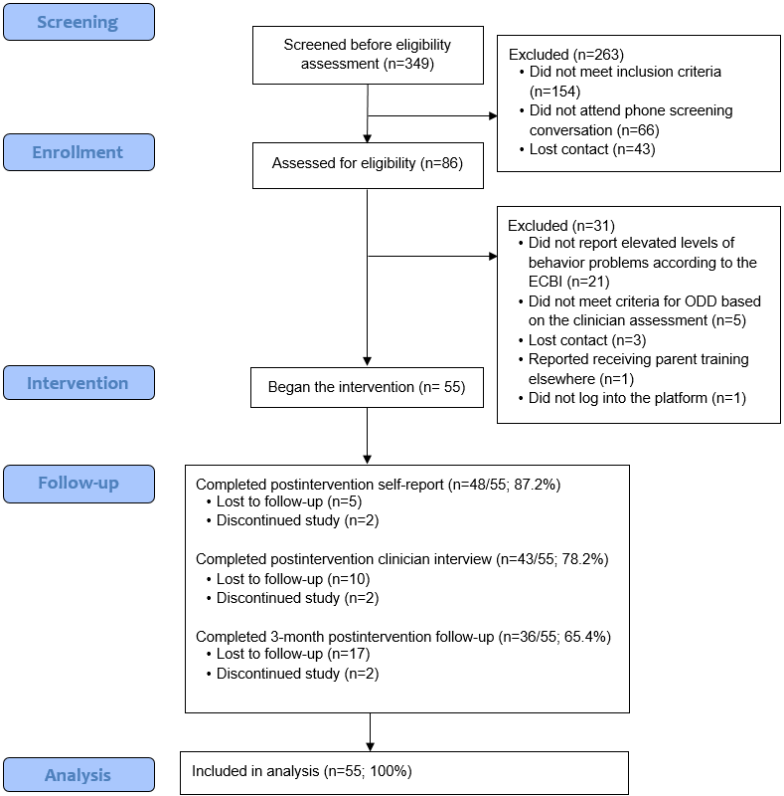
Flow of participants through the trial. ECBI Eyberg Child Behavior Inventory; ODD: oppositional defiant disorder.

The participant flow diagram is presented in Figure 1, and participant demographics are presented in Table 1. Overall, parents from 55 families with children with ODD enrolled in the study and began the intervention. Among them, 25/55 (46%) families began the intervention and completed their postintervention assessment before wartime, while the remaining 30/55 (54%) began the intervention and completed their postintervention assessment during wartime. The mean age of the children at the beginning of the intervention was 5.33 (SD 1.14) years, and 32/55 (58%) children were boys. The leading parent in all families was the mother, whose mean age was 36.7 (SD 3.54) years. No differences in demographic variables were identified based on intervention cohorts.

Parents in 34/55 families (62%) reported that this was the first intervention they had received for their child’s behavior problems. A chi-square test revealed a significant difference between those receiving the intervention for the first time and the rest of the sample in terms of religiosity (secular vs more religious; *χ*²_1_=4.34; *P*=.04). Among secular families, 14/27 (52%) reported that this was the first intervention they had received for their child’s behavioral problems, whereas among traditional or religious families, 22/28 (79%) reported the same. No other differences in demographic or outcome variables were found between these two categories.

It took parents 22.82 (SD 15.80) days on average to complete their onboarding procedure and begin the intervention. There was a significant difference in the time it took parents to begin the intervention based on study cohorts (*P*≤.004; ie, *P*=.004, *P*=.002, and *P*<.001). Specifically, during wartime, it took parents half the time (15.70 days) to begin the intervention than it did before wartime (31.36 days).

**Table 1:** Participant demographic characteristics and onboarding time.

				Total sample (N=55)		Before wartime (N=25)		During wartime (N=30)		Difference				
										Chi-square (*df*) or *Z*^a^			*P* value	
**Categorical variables, n (%)**														
	**Sex of the child**										0.09 (1)^b^			.76
		Male	32 (58)		14 (56)		18 (60)							
		Female	23 (42)		11 (44)		12 (40)							
	**Sex of the leading parent**										N/A^c^			N/A
		Female	55 (100)		25 (100)		30 (100)							
	**Who participates**										0.74 (1)^b^			.39
		Both parents	34 (62)		17 (68)		17 (57)							
		One parent	21 (38)		8 (32)		13 (43)							
	**Family structure^d^**										N/A			N/A
		Single	1(3)		0 (0)		1 (3)							
		Married	31 (84)		5 (71)		26 (87)							
		Cohabiting	3 (8)		1 (14)		2 (7)							
		Divorced	2 (5)		1 (14)		1 (3)							
		Widowed	0 (0)		0 (0)		0 (0)							
	**Education^e^**										0.10 (1)^b^			.75
		High school	10 (18)		5 (20)		5 (17)							
		Above	45 (82)		20 (80)		25 (83)							
	**House-level income^f^**										1.79 (2)^b^			.40
		<15,000	13 (24)		8 (32)		5 (17)							
		15,000-18,000	12 (22)		5 (20)		7 (23)							
		>18,000	30 (56)		12 (48)		18 (60)							
	**Religiosity**										2.86 (2)^b^			.24
		Secular	27 (49)		13 (52)		14 (47)							
		Traditional	16 (29)		9 (36)		7 (23)							
		Religious	12 (22)		3 (12)		9 (30)							
	**Weekly working hours^e^**										1.14 (3)^b^			.76
		Under 10	12 (22)		5 (20)		7 (23)							
		10-29	7 (13)		3 (12)		3 (12)							
		30-39	18 (33)		7 (28)		11 (37)							
		More than 39	18 (33)		10 (40)		8 (27)							
	**First intervention^g^**										0.87 (1)^b^			.35
		Yes	36 (66)		18 (72)		18 (60)							
		No	19 (35)		7 (28)		12 (40)							
**Continuous variables, mean (SD)**														
	Parent age (years)^e^			36.70 (3.54)		36.64 (3.30)		36.74 (3.78)		–0.43^a^			.67	
	Child age (years)			5.33 (1.14)		5.29 (1.04)		5.36 (1.24)		–0.26^a^			.79	
	Number of children in family			2.60 (.99)		2.80 (1.15)		2.40 (.81)		–1.46^a^			.14	
**Onboarding process**														
	Time from screening to intake completion (days)			20.07 (13.38)		26.48 (16.67)		14.73 (6.20)		–2.89^a^			.004^h^	
	Time from intake completion to first login (days)			2.74 (4.99)		4.88 (6.62)		0.97 (1.65)		–3.07^a^			.002^h^	
	Total time from screening to first login (days)			22.82 (15.80)		31.36 (17.33)		15.70 (6.62)		–3.53^a^			<.001^h^	

^a^The calculated standardized value (*Z*) of the Mann-Whitney *U* test.

^b^Chi-square test.

^c^N/A: not applicable.

^d^This question was accidentally not presented to the first 18 participants in the first cohort, and for this reason, we did not statistically examine the differences between the 2 cohorts in terms of this variable.

^e^Refers to the parent leading the intervention.

^f^In Israeli shekels (1 shekel was approximately US $3.8 at the time of the study).

^g^This was the first intervention the parents had received with the target of treating their child’s behavioral problems.

^h^Significant results.

### Program Usage and Completion

Program usage and completion rates are presented in Table 2. The mean time to complete the program was 12.34 (SD 5.18) weeks and included an average usage time of 138.6 (SD 63.41) minutes. Altogether, parents from 44/55 families (80%) completed the “overcoming disobedience” theme, and parents from 35/55 families (64%) completed the whole intervention. The total human support time invested on average per family was 38.81 (SD 35.32) minutes, most of which was spent on text messaging (mean time 30.2, SD 29.9 minutes).

Parents who completed the interventions during wartime logged into the program on significantly more days (*r*=.28; *P*=.04), and significantly more of them completed the “overcoming disobedience” theme (Cramer *V*=.27; *P*=.04). Parents from this cohort also received more messages from the human supporter (Cramer *V*=.28; *P*=.04). It is important to note that, during wartime, parental usage was higher on all metrics (eg, 70%, 21/30 program completers compared to 56%, 14/25 in the cohort before the war), although the results did not reach the level of statistical significance.

**Table 2:** Program completion and usage metrics.

Program usage			Total sample (N=55)		Before wartime (N=25)		During wartime (N=30)		Difference			
								*χ*^*2*^(*df*) or *Z*^a^		Cramer *V* or *r*^b^	*P* value	
**Categorical variable, n (%)**												
	“Overcoming disobedience” theme completers	44 (80)		17 (68)		27 (90)		4.12 (1)^c^		0.27^d^	.04^e^	
	Program completers	35 (63.6)		14 (56)		21 (70)		1.16 (1)^c^		0.14^d^	.28	
**Continuous variables, mean (SD)**												
	Number of login days	27.7 (12.73)		23.88 (13.11)		30.83 (11.70)		–2.07^a^		0.28^b^	.04^e^	
	Unique logins	31.4 (14.64)		28.20 (16.11)		34.03 (12.97)		–1.43^a^		0.19^b^	.15	
	Usage time (minutes)	138.6 (63.41)		120.28 (66.84)		148.30 (59.42)		–1.16^a^		0.16^b^	.24	
	Time using the program (weeks)	12.35 (5.18)		11.70 (6.38)		12.89 (3.96)		–1.24^a^		0.17^b^	.21	
**Human support, mean (SD)**												
	Messages sent by parents	5.90 (5.95)		6.12 (6.33)		5.67 (5.71)		–0.09^a^		0.01^b^	.93	
	Messages sent by the supporter	13.40 (4.93)		11.88 (4.80)		14.63 (4.77)		–2.07^a^		0.28^b^	.039^e^	
	Supporter time spent messaging (minutes)	30.20 (29.29)		35.44 (40.12)		25.87 (15.13)		–0.31^a^		0.04^b^	.75	
	Phone conversations	0.80 (0.92)		0.88 (1.09)		0.70 (0.75)		–0.28^a^		0.04^b^	.77	
	Supporter time spent in phone conversation (minutes)	8.60 (11.83)		8.84 (12.91)		8.40 (11.06)		–0.03^a^		0.004^b^	.97	
	Total human support time^f^	38.81 (35.32)		44.28 (48.17)		34.27 (18.83)		–0.13^a^		0.02^b^	.90	

^a^The Z statistic in this table represents the calculated standardized value of the Mann-Whitney *U* test.

^b^Represents the respective effect size.

^c^Chi-square test.

^d^Cramer *V*.

^e^Significant results.

^f^Includes the time it took the supporter to write messages, document notes, and discuss cases with the supervisor.

### Intervention Outcomes

Descriptive statistics and statistical differences in self-reported changes in child behaviors and parenting variables at postintervention and follow-up time points are presented in Table 3. Statistical differences in self-reported changes between the 2 cohorts are presented in Table 4. Significant improvements were found among all outcome variables postintervention (compared to baseline), with large effect size improvements in child behavior problems (ECBI Cohen *d*=0.79-0.83), medium to large effect size improvements in parenting styles (PS Cohen *d*=0.52-0.87), and medium effect size improvements in task-specific sense of efficacy (PTC Cohen *d*=0.57-0.65) and positive parenting practices (APQ Cohen *d*=0.39). Effects remained significant at follow-up time points for all outcome variables, with descriptively larger effect sizes than postintervention (eg, ECBI *Cohen* *d*=1.02-1.10). No differences were found between the 2 cohorts in terms of self-reported variables.

Changes in cases not meeting the ODD criteria postintervention based on the clinician interviews are presented in Table 5. The impact of wartime on not meeting the criteria for ODD postintervention was found to be marginally significant (*P*=.07). In the prewartime cohort, 17/25 children (68%) did not meet the criteria for ODD following the intervention; during wartime, 13/30 children (43%) did not meet the criteria for ODD following the intervention.

**Table 3:** Descriptive statistics and differences in reported changes from baseline to postintervention and follow-up time points.

Measures		Baseline	Postintervention	Follow-up^a^	Baseline to postintervention				Baseline to follow-up		
					*t* test (*df*) or *Z*^b^	Cohen *d* or *r*^c^	*P* value	*t* test (*df*) or *Z*^b^		Cohen *d* or *r*^c^	*P* value
**Total (N=55), mean (SD)**											
	ECBI^b^ Intensity	164.16 (20.56)	138.75 (28.78)	132.88 (23.91)	6.12 (54)^e^	0.83^f^	<.001^g^	8.16 (54)		1.10	<.001^g^
	ECBI Problems	23.96 (4.54)	17.40 (8.03)	15.89 (7.08)	5.83 (54)^e^	0.79^f^	<.001^g^	7.55 (54)		1.02	<.001^g^
	PS^h^ Laxness	3.45 (1.19)	2.96 (1.06)	2.76 (.74)	3.88 (54)^e^	0.52^f^	<.001^g^	4.62 (54)		0.62	<.001^g^
	PS Overreactivity	3.55 (.68)	2.81 (1.00)	2.73 (.78)	6.43 (54)^e^	0.87^f^	<.001^g^	8.52 (54)		1.15	<.001^g^
	PTC^i^ Setting	64.33 (14.76)	75.92 (16.57)	78.32 (12.33)	–4.82 (54)^e^	–0.65^f^	<.001^g^	–6.07 (54)		–0.82	<.001^g^
	PTC Behavioral	49.42 (24.11)	65.05 (22.88)	72.30 (15.51)	–4.20 (54)^e^	–0.57^f^	<.001^g^	–6.81 (54)		–0.92	<.001^g^
	APQ^j^	4.30 (.45)	4.46 (.40)	4.45 (.32)	–2.86 (54)^e^	–0.39^f^	.006^g^	–2.69 (54)		–0.36	.01^g^
**Before wartime (n=25), mean (SD)**											
	ECBI Intensity	165.08 (22.78)	136.39 (30.02)	129.02 (24.05)	–3.50	0.70	<.001^g^	–4.08		0.81	<.001^g^
	ECBI Problems	23.40 (4.44)	17.09 (6.80)	14.48 (6.41)	–3.13	0.63	.002^g^	–3.84		0.70	<.001^g^
	PS Laxness	3.36 (1.16)	2.81 (1.08)	2.64 (0.64)	–2.22	0.44	.03^g^	–2.52		0.50	.01^g^
	PS Overreactivity	3.70 (0.65)	2.96 (1.07)	3.03 (0.73)	–3.34	0.67	<.001^g^	–3.47		0.69	<.001^g^
	PTC Setting	64.30 (16.23)	75.03 (17.00)	78.40 (10.71)	–2.50	0.50	.01^g^	–3.54		0.70	<.001^g^
	PTC Behavioral	44.72 (21.94)	65.92 (21.48)	72.08 (16.60)	–3.46	0.70	<.001^g^	–4.18		0.84	<.001^g^
	APQ	4.43 (0.43)	4.48 (0.36)	4.50 (0.34)	–.87	0.17	.39	–.87		0.17	.39
**During wartime (n=30), mean (SD)**											
	ECBI Intensity	163.40 (18.87)	140.72 (28.07)	136.10 (23.70)	–3.75	0.68	<.001^g^	–4.47		0.82	<.001^g^
	ECBI Problems	24.43 (4.64)	17.65 (9.03)	17.07 (7.49)	–3.67	0.67	<.001^g^	–4.06		0.67	<.001^g^
	PS Laxness	3.54 (1.22)	3.09 (1.05)	2.86 (0.80)	–2.60	0.47	.009^g^	–3.08		0.56	.002^g^
	PS Overreactivity	3.43 (0.97)	2.69 (0.95)	2.46 (0.73)	–4.00	0.73	<.001^g^	–4.44		0.81	<.001^g^
	PTC Setting	64.36 (13.70)	76.66 (16.46)	78.25 (13.72)	–2.95	0.54	.003^g^	–3.63		0.66	<.001^g^
	PTC Behavioral	53.33 (25.31)	64.32 (24.31)	72.48 (14.82)	–1.91	0.35	.06	–3.22		0.59	.001^g^
	APQ	4.20 (0.45)	4.43 (0.43)	4.40 (0.29)	–2.71	0.49	.007^g^	–2.21		0.40	.03^g^

^a^The 3-month postintervention follow-up.

^b^The *Z* statistic in this table represents the calculated standardized value of the Wilcoxon test.

^c^Represents the respective effect size.

^d^ECBI: Eyberg Child Behavior Inventory.

^e^*t* test.

^f^Cohen d.

^g^Significant results.

^h^PS: Parenting Scale.

^i^PTC Behavioral: Parenting Tasks Checklist.

^j^APQ: Alabama Parenting Questionnaire, Positive Parenting Practices Subscale.

**Table 4:** Statistical differences in reported changes between the 2 cohorts.

Measures	Baseline: postreported change							Baseline: follow-up–reported change					
	Before wartime (n=25)	During wartime (n=30)	Differences				Before wartime (n=25)		During wartime (n=30)		Differences		
	Mean (SD)	Mean (SD)	*Z* ^a^	*r*	*P* value	Mean (SD)				Mean (SD)	*Z*	*r*	*P* value
ECBI^b^ Intensity	–28.70 (32.43)	–22.68 (29.61)	–.84	.11	.40	–36.06 (29.27)				–27.30 (27.59)	–1.43	.19	.15
ECBI Problems	–6.31 (7.82)	–6.78 (8.89)	–.33	.04	.74	–8.91 (7.62)				–7.36 (8.24)	–1.23	.17	.22
PS^c^ Laxness	–.54 (.95)	–.46 (.83)	–.19	.03	.85	–.71 (1.22)				–.68 (1.04)	–.042	.06	.97
PS Overreactivity	–.74 (1.09)	–.74 (.77)	–.21	.03	.83	–.66 (.70)				–.97 (.72)	–1.31	.18	.19
PTC^d^ Setting	10.72 (18.24)	12.30 (17.72)	–.51	.07	.61	14.09 (15.83)				13.89 (18.31)	–.41	.06	.69
PTC Behavioral	21.20 (22.28)	10.98 (30.85)	–1.45	.20	.15	27.30 (21.79)				19.14 (27.03)	–1.36	.18	.17
APQ^e^	.06 (.36)	.24 (.41)	–1.41	.19	.16	.08 (.35)				.21 (.45)	–1.36	.18	.17

^a^The Z statistic in this table represents the calculated standardized value of the Wilcoxon test (*r* represents the respective effect size).

^b^ECBI: Eyberg Child Behavior Inventory.

^c^PS: Parenting Scale.

^d^PTC Behavioral: Parenting Tasks Checklist.

^e^APQ: Alabama Parenting Questionnaire, Positive Parenting Practices Subscale.

**Table 5:** Postintervention changes in meeting oppositional defiant disorder criteria based on clinician assessment.

Cohort		Number of symptoms preintervention	Number of symptoms postintervention	Cases not meeting ODD^a^ criteria postintervention	Difference between the cohorts in the ODD criteria change	
		Mean (SD)	Mean (SD)	n (%)	Chi-square (*df*)	*P* value
**Total (N=55)**		5.20 (.99)	3.04 (1.87)	30 (55)	3.35 (1)	.07
	Before wartime (n=25)	5.24 (1.20)	2.52 (1.54)	17 (68)		
	During wartime (n=30)	5.17 (.80)	3.47 (2.03)	13 (43)		

^a^ODD: oppositional defiant disorder

## Discussion

### Principal Findings

This study found that the DPT primarily reached parents who had not received care for the current condition. The demographic characteristics of our sample, particularly regarding education, income, and religiosity, were comparable to national data for parents in Israel, supporting the representativeness of the study population [30-32]. In addition, the DPT was found to be effective at reducing child behavior problems and improving parenting-related variables. Differences were identified between the study cohorts based on the context of war in the region.

This study found that the remote recruitment procedure was successful at reaching mostly parents who had never received an intervention for their child’s ODD (34/55, 62%). This finding aligns with broader research showing that digital and remote mental health interventions can indeed increase access and reach new people in need, or earlier in the prodromal process [33,34]. It is important to note that this study also found that we were able to reach more families for the first time (22/28, 79%) if they were nonsecular. It could be that, in Israel, secular families are more likely to seek psychological services independently, but this needs to be further examined in future studies.

In terms of program usage, most parents (44/55, 80%) completed the “overcoming disobedience” theme, the most important skill area in the program. Parents also used the program for more than 2 hours (138.6 minutes) on average, scattered across multiple sessions (a total of 31.4 unique logins). This pattern of use differs completely from that of therapist-led parent training, which is based on a much smaller number of sessions, each lasting 40-50 minutes [35]. It could be that this pattern of use (ie, in small pockets of available time) is more relevant to parents who decide to use digital interventions to self-manage their child’s state. The total human support time spent during this program was quite small (an average of 38.81 minutes), with parents preferring to message the supporter rather than engage in phone conversations. It is worth noting that studies in which support is primarily provided through phone calls have reported a higher level of human support (eg, [10,36]). It would be interesting to further investigate the extent to which parents prefer phone calls when text messaging is available and how this choice influences the outcomes.

In terms of wartime impact, the results showed that, during wartime, parents completed their onboarding process in half the time it took before the war. Parents also used the program more extensively, with 90% (27/30) completing the “overcoming disobedience” skill acquisition phase and logging into the program 7 more days than the cohort participating before wartime. The greater volume of supporter messages observed during wartime is best interpreted as a protocol-triggered outreach message based on a higher share of parents reaching key phases in the program (eg, “overcoming disobedience”); notably, parent-initiated messages were similar across cohorts, and total support time did not differ. This usage pattern fits with the patterns described by Sakari et al [13] in comparing the use of a DPT before and during the COVID-19 pandemic. That report indicated that program completion rates actually increased during the COVID-19 epidemic. It is plausible that the growing need for support during times of crisis is manifested in increased use of the digital intervention. However, while our findings indicated comparable improvements based on parental self-reports, our study revealed a close to significant difference between the 2 cohorts in the percentage of children not meeting the criteria for ODD post intervention based on clinician assessment (13/30, 43% recovered during wartime vs 17/25, 68% before the war). Although this is only a marginally significant result, it suggests a potential trend toward an effect, mainly because of the descriptively large difference in percentages of children meeting the ODD criteria.

Research has indicated that environmental stressors, such as those present during wartime, can significantly affect the efficacy of mental health interventions. For instance, studies have shown that ongoing stress and trauma can complicate the treatment process (eg, [37]). Supporting this notion, during the war that began on October 7, 2023, a study examined the prevalence of mental illness and reported prevalence rates of generalized anxiety disorder and depression of over 42% in an Israeli representative sample [12]. In our study, it could be that the ability of parents to use coping strategies was strained, thereby impacting the overall effectiveness of the parent training intervention. It is also possible that parents’ own mental health, particularly symptoms of anxiety and depression, played a role in both their engagement with and response to the intervention during wartime. Elevated distress may have limited parents’ capacity to apply therapeutic strategies consistently, potentially contributing to the lower clinician-rated recovery rates observed in this period. However, we did not have any relevant data to assess whether parental mental health impacted both the increased usage and decreased effectiveness. Future studies could benefit from incorporating brief parent mental health screeners to examine how parental anxiety and depression relate to engagement patterns and intervention outcomes, as well as whether improvements in the parent–child relationship lead to secondary benefits in parental well-being.

While this paper was not designed as a cost-benefit analysis, cost and clinician time are relevant factors when considering the scalability of an intervention and are therefore worth noting. The direct cost per treatment in this study included 2 clinician assessments (30 minutes each, before and after the intervention) and an average of less than an hour of human support during the intervention. Regarding the latter, the supporter spent less than 40 minutes on average providing support, including note-taking. Assuming a cost of approximately Israeli Shekels (ILS) 200 per clinician assessment and ILS 100 per hour of human support (including overheads), the direct cost per family is estimated at ILS 500 (≈US $130; approximately ILS 1=US $3.8 at the time of the study). These estimates exclude costs related to upgrading and maintaining the platform, office needs, administrative tasks, and management. Overall, the time and cost required for this guided DPT are considerably lower than those reported for traditional behavior parent training programs, underscoring the model’s scalability potential.

### Limitations

This study implemented a 1-arm intervention condition without a control group. It was not feasible to deploy a no-treatment control condition while examining the real-world use of an intervention, as the recruitment and promotion of the intervention (and study) would have differed. An ethical consideration also arises during wartime, in that it would not be ethical to withhold a timely digital intervention when people may need it the most. The absence of a control condition means that the overall pre- to postintervention effects could be attributed to additional factors. However, the reported effects are similar to those reported in a randomized controlled pilot trial of the same DPT in its unguided format [11]. Another limitation concerns our operationalization of engagement, which focused on objective indicators of usage such as log data, completion rates, duration, and navigation patterns. While these measures are commonly used and provide valuable insights into user behavior, they do not capture the full spectrum of engagement [38,39]. Future studies should complement such objective measures with indicators of experience, including satisfaction, engagement in therapeutic activities, affective responses, and attention. Integrating both behavioral and experiential dimensions of engagement would allow a more comprehensive understanding of how parents interact with digital interventions and how engagement relates to outcomes.

### Conclusions

DPTs present a form of care that could reach parents who have not sought treatment through traditional channels. These programs are acceptable, with preliminary evidence showing them to be highly used and effective. Examining the impact of DPTs during crisis times provides valuable insights into the potential use of these scalable interventions, which have a particular advantage in terms of deployment during crises. Stakeholders should have an articulated plan for the deployment of such interventions. Such strategic planning can not only address immediate needs during a crisis but also contribute to long-term public health strategies by integrating DPTs as a regular component of mental health care provision. Future research should explore ways to further optimize human support models, assess the role of parental mental health in engagement and outcomes, and evaluate the program’s scalability across diverse real-world settings.

## Appendix

Multimedia Appendix 1 A detailed review of Benevolent Parenting Intervention.

Multimedia Appendix 2 Completers' sample—descriptive statistics and differences in reported changes between baseline and postintervention time points.

Multimedia Appendix 3 Completer's sample—postintervention changes in meeting oppositional defiant disorder criteria based on clinician assessment.

## Abbreviations

APQ: Alabama Parenting Questionnaire
DPT: Digital Parent Training
ECBI: Eyberg Child Behavior Inventory
ILS: Israeli Shekels
ODD: oppositional defiant disorder.
PS: Parenting Scale
PTC: Parenting Tasks Checklist

## Data Availability

The datasets generated or analyzed during this study are available from the corresponding author on reasonable request.
