# Supplementary material for: Evaluating the Reach, Usage, Human Support Needs, and Clinical Outcomes of Digital Parent Training for Child Oppositional Defiant Disorder Before and During Wartime: Longitudinal Study
Source: JMIR Pediatr Parent. 2025 Dec 22;8:e80420. doi: 10.2196/80420 (PMC12770920; doi:10.2196/80420)
Supplement: Multimedia Appendix 1 [file pediatrics_v8i1e80420_app1.docx]

A detailed review of Benevolent Parenting Intervention

*2.2.1 Content.* Benevolent parenting incorporates common components of evidence-based DPTs (e.g. for childhood disruptive behaviors (Baumel, Pawar, et al., 2017; Sanders et al., 2012; Sourander et al., 2016)). The program includes seven themes that are recommended to be completed within a two-month time period; each theme involves a specific subject area: (1) introduction to parent training aimed at treating child behavior problems; (2) quality time and positive interactions; (3) parental emotion regulation; (4) effective routines and clear ground rules; (5) recognizing positive behaviors/ignoring minor negative behaviors; (6) overcoming disobedience; and (7) mindful parenting and conversation between partners. Three themes (#2, #3, #5) are not mandatory and are recommended to parents based on their answers to a brief questionnaire completed during the first theme. Parents can then decide if they would like to address these additional themes in their personal program. The order of themes can also be adjusted based on the parent’s situation. For instance, for parents who report extreme tension at home, themes 5 and 6 (the “overcoming disobedience” sequence) can move to the beginning.

The program is based on pre-defined decision rules that are either event- or time-based (Muench & Baumel, 2017). This feature is meant to increase user engagement through effort optimization design (for a review, see (Baumel & Muench, 2021)) and therapeutic persuasiveness (for a review, see (Baumel, Faber, et al., 2017; Baumel & Yom-Tov, 2018)). Each theme in the program comprises a short *learning phase* followed by a 1–2 week *focusing phase*.

The learning phase takes 10–20 minutes to complete and includes videos, audio, pictures, and texts, as well as interactive features, such as multiple-choice questions with direct feedback and the ability to easily navigate between module chapters. The focusing phase is designed to help the desired therapeutic activities become salient in the parents’ mind and to help them to acquire skills in a non-judgmental manner, while avoiding the burden and potential failures that may be associated with the idea of “training”. For that reason, the program incorporates the following features:

1. Call to action: During each focusing phase, parents receive timely digital triggers (tips, motivation) related to the specific goals and therapeutic activities of the modules they have completed.
2. Creation of salience through self-monitoring and ongoing feedback: Specific parental practices related to the therapeutic activities/skills currently being taught are documented within the system using a brief daily report that includes no more than seven logic-driven multiple-choice questions and takes less than one minute to complete (see Figure I for an example).


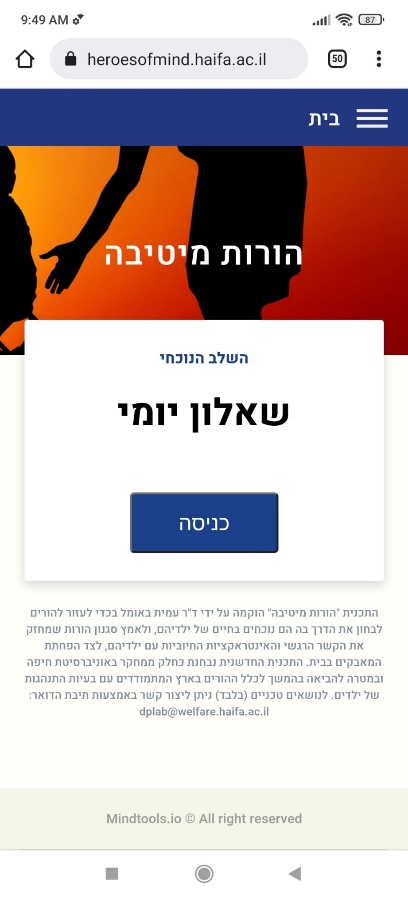

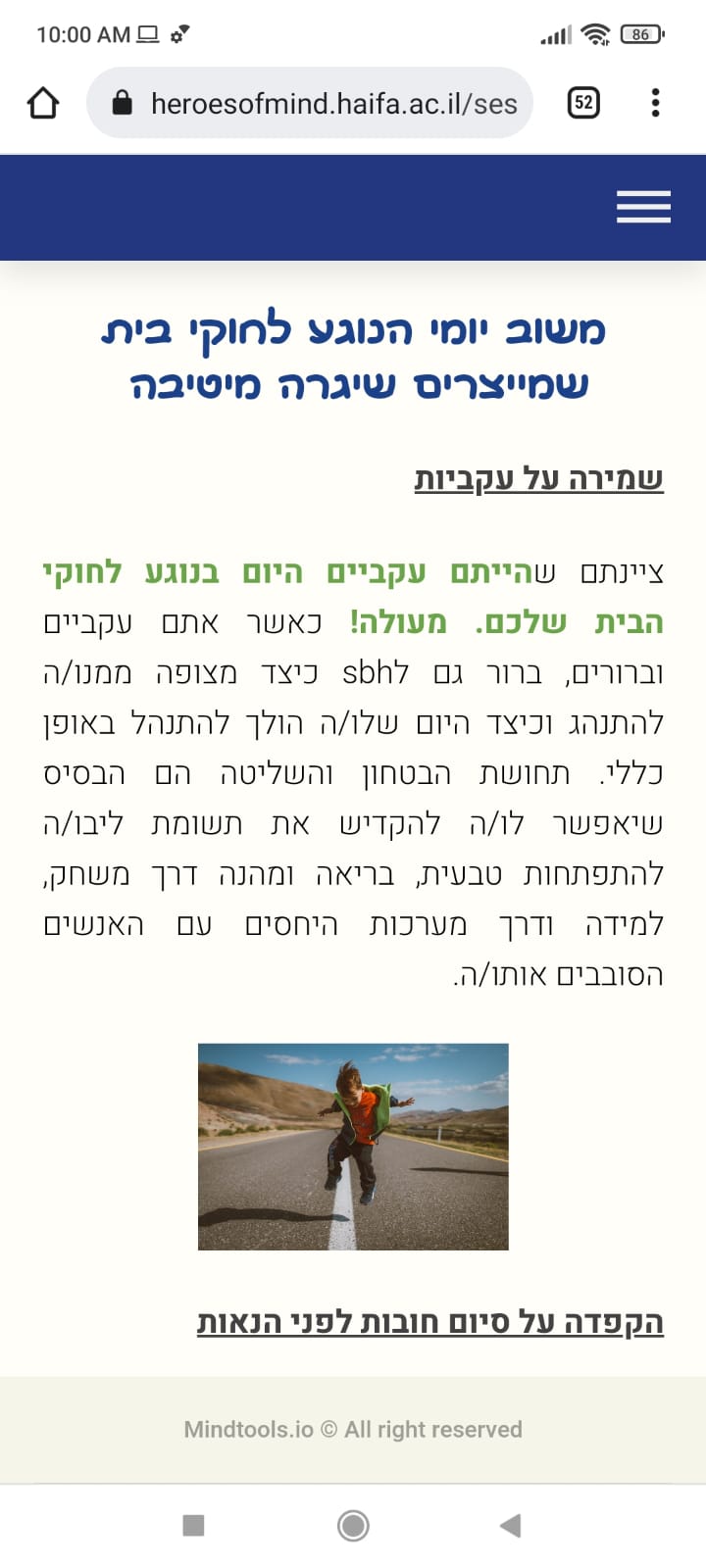


**Figure S1.** Mobile view for entering a daily brief self-monitoring questionnaire (left-screen) and the personalized daily positive feedback (right-screen).

1. Adaptation to user state: Parents’ reports on their activities are used to acknowledge their success and to suggest additional actions based on the specific goals identified. Effort related to desired therapeutic activities is adaptive, based on our previous work on adaptive interventions (e.g. (Muench et al., 2017)) and the research literature (Krebs et al., 2010). This work has demonstrated that programs that adapt to the recipient’s progress outperform static interventions.

The platform includes an enhanced e-learning user interface, a human support chat-based component that enables secure text-based asynchronous communication between the participant and the supporter, the ability to send personalized triggers (notifications) to users, and content management tools that enable the provision of logic-driven interventions (see Figure II for an example).


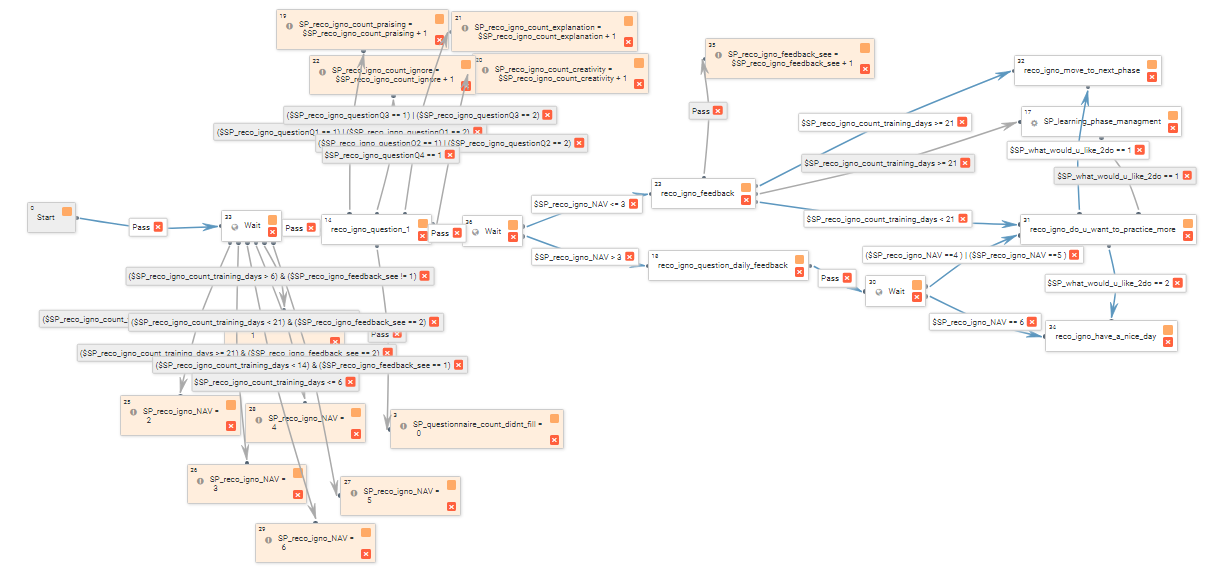


**Figure S2.** An example of the system’s admin user interface that enables the creation of a logic-driven intervention. Within each arrow, a condition can be created that determines whether a certain node will be deployed. Blue arrows are between webpages/notifications that the participants view and interact with; gray arrows are back-end processes.

*2.2.2 Human Support.* Human support followed a manual based on our previous work (Baumel et al., 2016; Baumel et al., 2018) and on the supportive accountability (Mohr et al., 2011) and efficiency (Schueller et al., 2016) models. The support aimed at encouraging participants to use the program and apply their skills and therapeutic goals in daily life, and at problem solving. Support began with an initial message sent when parents first logged into the program. This message set expectations for the supporter–parent relationship, ensured that the program was properly accessible, and included an invitation to schedule a phone conversation (although this was not mandatory). After the initial engagement procedure, parents received one message following the completion of each learning and focusing phase. These messages aimed at supporting parental accountability, acknowledging the work done so far, and encouraging the application of learned skills. The supporter also contacted parents via the messaging platform if disengagement was identified (i.e. when a parent did not complete a learning phase within a week or did not complete a questionnaire during the focusing phase for five consecutive days). The supporter responded to all participant-initiated messages within one working day. In addition, the supporter offered, but did not require, a phone conversation following the completion of the “overcoming disobedience” module to reinforce parental efforts during the subsequent focusing phase. The supporter — an educational psychologist trained and supervised by the study's principal investigator (PI)—used a dashboard that provided information on engagement markers and participants' progress.

References

23. Baumel, A., Correll, C. U., Hauser, M., Brunette, M., Rotondi, A., Ben-Zeev, D., . . . Kane, J. M. (2016). Health technology intervention after hospitalization for schizophrenia: Service utilization and user satisfaction. *Psychiatric Services*, *67*(9), 1035-1038. https://doi.org/10.1176/appi.ps.201500317

19. Baumel, A., Faber, K., Mathur, N., Kane, J. M., & Muench, F. (2017). Enlight: A comprehensive quality and therapeutic potential evaluation tool for mobile and web-based eHealth interventions [Original Paper]. *Journal of Medical Internet Research*, *19*(3), e82. https://doi.org/10.2196/jmir.7270

18. Baumel, A., & Muench, F. J. (2021). Effort-Optimized Intervention Model: Framework for Building and Analyzing Digital Interventions That Require Minimal Effort for Health-Related Gains. *Journal of Medical Internet Research*, *23*(3), e24905.

6. Baumel, A., Pawar, A., Mathur, N., Kane, J. M., & Correll, C. U. (2017). Technology-assisted parent training programs for children and adolescents with disruptive behaviors: A systematic review. *Journal of Clinical Psychiatry*, *78*(8), e957-e969. https://doi.org/10.4088/JCP.16r11063

22. Baumel, A., Tinkelman, A., Mathur, N., & Kane, J. M. (2018). Digital peer-support platform (7Cups) as an adjunct treatment for women with postpartum depression: Feasibility, acceptability, and preliminary efficacy study. *JMIR mHealth and uHealth*, *6*(2), e38. https://doi.org/10.2196/mhealth.9482

20. Baumel, A., & Yom-Tov, E. (2018). Predicting user adherence to behavioral eHealth interventions in the real world: Examining which aspects of intervention design matter most. *Translational Behavioral Medicine*, *5*(5), 793-798. https://doi.org/10.1093/tbm/ibx037

Krebs, P., Prochaska, J. O., & Rossi, J. S. (2010). A meta-analysis of computer-tailored interventions for health behavior change. *Preventive Medicine*, *51*(3-4), 214-221.

Mohr, D. C., Cuijpers, P., & Lehman, K. (2011). Supportive accountability: A model for providing human support to enhance adherence to eHealth interventions [Viewpoint]. *Journal of Medical Internet Research*, *13*(1), e30. https://doi.org/10.2196/jmir.1602

17. Muench, F., & Baumel, A. (2017). More than a text message: Dismantling digital triggers to curate behavior change in patient centered health interventions. *Journal of Medical Internet Research*, *19*(5), e147. https://doi.org/10.2196/jmir.7463

Muench, F., van Stolk-Cooke, K., Kuerbis, A., Stadler, G., Baumel, A., Shao, S., . . . Morgenstern, J. (2017). A randomized controlled pilot trial of different mobile messaging interventions for problem drinking compared to weekly drink tracking. *PloS One*, *12*(2), e0167900.

Sanders, M. R., Baker, S., & Turner, K. M. (2012). A randomized controlled trial evaluating the efficacy of Triple P Online with parents of children with early-onset conduct problems. *Behaviour Research and Therapy*, *50*(11), 675-684.

Schueller, S. M., Tomasino, K. N., & Mohr, D. C. (2016). Integrating human support into behavioral intervention technologies: The efficiency model of support. *Clinical Psychology: Science and Practice*.

37. Sourander, A., McGrath, P. J., Ristkari, T., Cunningham, C., Huttunen, J., Lingley-Pottie, P., . . . Sinokki, A. (2016). Internet-assisted parent training intervention for disruptive behavior in 4-year-old children: A randomized clinical trial. *JAMA psychiatry*, *73*(4), 378-387.
