# Supplementary material for: Evaluating the Reach, Usage, Human Support Needs, and Clinical Outcomes of Digital Parent Training for Child Oppositional Defiant Disorder Before and During Wartime: Longitudinal Study
Source: JMIR Pediatr Parent. 2025 Dec 22;8:e80420. doi: 10.2196/80420 (PMC12770920; doi:10.2196/80420)
Supplement: Multimedia Appendix 2 [file pediatrics_v8i1e80420_app2.docx]

**Table S1.** Completers' sample—descriptive statistics and differences in reported changes between baseline and postintervention time points.

|  | Baseline (N=55) | Post-intervention  (N=48) | Follow-up  (N=36) | Baseline to post | | | Baseline to follow-up | | |
| --- | --- | --- | --- | --- | --- | --- | --- | --- | --- |
| Measure | M (SD) | M (SD) | M (SD) | *t* (47) | *p* | *d ^a^* | *t* (35) | *p* | *d ^a^* |
| ECBI Intensity | 164.16  (20.56) | 138.85  (30.84) | 132.97 (29.63) | 5.64 | **<.001** | 0.82 | 5.89 | **<.001** | 0.98 |
| ECBI Problems | 23.96 (4.54) | 17.40 (8.59) | 15.94 (8.75) | 5.53 | **<.001** | 0.80 | 5.13 | **<.001** | 0.89 |
| PS Laxness | 3.45 (1.19) | 2.94 (1.13) | 2.75 (.87) | 3.77 | **<.001** | 0.54 | 3.20 | **.003** | 0.53 |
| PS Over-reactivity | 3.55 (.68) | 2.81 (1.07) | 2.82 (.91) | 6.06 | **<.001** | 0.87 | 6.28 | **<.001** | 1.05 |
| PTC Setting | 64.33 (14.76) | 75.85 (17.74) | 78.43 (15.27) | -4.11 | **<.001** | -0.59 | -4.03 | **<.001** | -0.67 |
| PTC Behavioral | 49.42 (24.11) | 65.13 (24.51) | 72.49 19.22 | -3.62 | **<.001** | -0.52 | -5.13 | **<.001** | -0.85 |
| APQ | 4.30 (.45) | 4.48 (.41) | 4.44 (.39) | -2.59 | **.013** | **-0.37** | -1.54 | .134 | -0.26 |

*Note.* Significant results are in bold. *^a^* Cohen’s *d.*
