# Supplementary material for: Evaluating the Reach, Usage, Human Support Needs, and Clinical Outcomes of Digital Parent Training for Child Oppositional Defiant Disorder Before and During Wartime: Longitudinal Study
Source: JMIR Pediatr Parent. 2025 Dec 22;8:e80420. doi: 10.2196/80420 (PMC12770920; doi:10.2196/80420)
Supplement: Multimedia Appendix 3 [file pediatrics_v8i1e80420_app3.docx]

**Table S1.** Completer sample—postintervention changes in meeting oppositional defiant disorder criteria based on clinician assessment.

|  | Number of symptoms  pre-intervention | | Number of symptoms  post-intervention | | Cases not meeting ODD criteria post-intervention | Difference between the cohorts in ODD criteria change | |
| --- | --- | --- | --- | --- | --- | --- | --- |
| Cohort | N | M (SD) | N | M (SD) |  | *χ^2^*(1) | *p* |
| Total | 55 | 5.20 (.99) | 43 | 3.05 (2.10) | 51.2% (22/43) | 3.15^a^ | .076 |
| Before wartime | 25 | 5.24 (1.20) | 16 | 2.25 (1.84) | 68.8% (11/16) |  |  |
| During wartime | 30 | 5.17 (.80) | 27 | 3.52 (2.14) | 40.7% (11/27) |  |  |
